# The Maternal Milk Microbiome in Mammals of Different Types and Its Potential Role in the Neonatal Gut Microbiota Composition

**DOI:** 10.3390/ani11123349

**Published:** 2021-11-23

**Authors:** Yile Ge, Wei Zhu, Lu Chen, Diyan Li, Qingqing Li, Hang Jie

**Affiliations:** 1 Farm Animal Genetic Resources Exploration and Innovation Key Laboratory of Sichuan Province, Sichuan Agricultural University, Chengdu 611130, China; 2019202021@stu.sicau.edu.cn (Y.G.); weizhu451@163.com (W.Z.); 2020202014@stu.sicau.edu.cn (L.C.); diyanli@sicau.edu.cn (D.L.); 2 Life Science College, Southwest Forestry University, Kunming 650224, China; 3 Kunming Xianghao Technology Co., Ltd., Kunming 650204, China; 4 Bio-Resource Research and Utilization Joint Key Laboratory of Sichuan and Chongqing, Chongqing Institute of Medicinal Plant Cultivation, Chongqing 408435, China

**Keywords:** maternal milk, neonate, gut microbiota, functional prediction, source tracking

## Abstract

**Simple Summary:**

In order to find the effects of host diet and phylogeny on maternal milk microbes and the contribution of the maternal milk microbiota to the neonatal gut microbiota, nine species of mammals of three type groups (herbivore, omnivore, and carnivore) were selected in this study. Our results showed that different types of animals and phylogeny factors may have driven the microbiota of mammalian maternal milk. Source-tracking analysis showed that the contributions of bacteria from maternal milk to the microbiota of neonates of different animals were different at day 3 after neonatal birth.

**Abstract:**

Maternal milk, a main source of nutrition for neonates in early life, has attracted attention. An increasing number of studies have found that maternal milk has a high microbial diversity, as well as factors that might influence this diversity. However, there is a lack of knowledge regarding the effects of host diet and phylogeny on maternal milk microbes and the contribution of the maternal milk microbiota to the neonatal gut microbiota. Here, we analyzed the maternal milk and fecal microbiota of nine species (lion, dog, panda, human, mouse, rhesus macaque, cow, goat, and rabbit) of mammals of three type groups (herbivore, omnivore, and carnivore) using 16S rRNA amplicon sequencing. Our study provided evidence of host diet and phylogeny on the maternal milk microbiota. Moreover, functional prediction revealed that the carnivores had a significantly higher percentage of base excision repair, glycerolipid metabolism, taurine and hypotaurine metabolism, inorganic ion transport and metabolism, and nucleotide metabolism; while arginine and proline metabolism showed enrichment in the herbivore group. Source-tracking analysis showed that the contributions of bacteria from maternal milk to the microbiota of neonates of different mammals were different at day 3 after neonatal birth. Overall, our findings provided a theoretical basis for the maternal milk microbiota to affect neonatal fecal microbiota at day 3 after neonatal birth.

## 1. Introduction

The mammalian gut microbiota is believed to promote key biological activities through interactions with the digestive [1], immune [2], and nervous systems of its host [3]. Previous studies have suggested that the diversity, structure, and function of the mammalian gut microbiome are mainly shaped by adaptation to host diet [4] and phylogeny [5]. *Bacteroidales*, *Clostridiales*, *Fibrobacteriales*, and *Spirochaetales*, which are highly diverse cellulolytic obligate anaerobes, colonize most herbivore guts, promoting microbial fermentation to increase nutrient absorption. In contrast, the gut microbiota of omnivores and carnivores are dominated by the facultative anaerobes *Enterobacteriaceae* and *Enterococcus* [6,7].

Maternal milk is a primary source of nutrition for neonates in early life and contains many abundant nutrients, including protein, fat, sugar, etc. [8,9]. Recent studies have found that milk contains a large number of microbes, with estimates ranging from 10–10^5^ [10]. An increasing number of studies have found that maternal milk microbes are crucial in guiding the spatiotemporal development of the microbiota in the neonatal gut, and aid in defense against pathogens before the immune system has matured [2,11,12,13]. In mammals, the initial colonists of the newborn gut microbes originate from the maternal placenta [14] and vaginal [15]. Postnatal factors include antibiotic treatment, breast milk, or environmental exposure [16]. Recent studies have found that microbes, such as *Bifdobacterium* [17], *Lactobacillus plantarum* [18], and other beneficial microorganisms, can be transmitted vertically to the infant gut during breastfeeding. In addition, transmission of *Escherichia coli* [19], *Streptococcus, Veillonella, Rothia*, and other microbes has also been reported [20]. Therefore, understanding the source of microbes in maternal milk and the factors that affect them provides an exciting opportunity to improve the health of neonates [21].

The source of microbes in maternal milk is unclear. One hypothesis of an entero-mammary route of transmission has been proposed [22]. This hypothesis was supported by later studies [23,24]. The host diet is a main determinant of gut microbial diversity [4]. Whether the host diet affects breast microbiome populations is unknown. However, Shively et al. found that mammary gland microbiome could be modulated by a dietary intervention, suggesting ongoing bacterial translocation from the gastrointestinal tract to the mammary gland [25]. A large number of studies are needed to explain whether the host diet affects the breast microbiome. In this study, we performed high-throughput sequencing of 16S rRNA gene amplicons from the maternal milk microbes and feces microbes of nine mammalian species. We aimed to characterize the bacterial community in the maternal milk of mammals of three type groups (herbivore, omnivore, and carnivore) and to identify the significant abundant bacterial taxa in response to the different animals. In addition, we also assessed the relative contributions of microbes from the maternal milk to the feces microbiomes of neonates.

## 2. Materials and Methods

### 2.1. Sample Collection

At the initial study visit, we collected recent data (including age, physical condition, diet, and antibiotics), and infant feeding characteristics (breastfeeding, formula, solid foods, and antibiotics). We collected 71 samples from nine mammalian species, including 35 fresh fecal samples and 36 fresh maternal milk samples (see Table S1 in the Supplementary Materials). All neonates’ fresh fecal samples were collected at day 3 after neonatal birth. The fresh fecal and milk samples of giant panda (*Ailuropoda melanoleuca*), lion (*Panthera leo*), goat (*Capra hircus*), and rhesus macaque (*Macaca mulatta*) were collected at the Chengdu Zoo. The samples of fresh fecal and milk from dog (*Canis lupus familiaris*), mouse (*Mus musculus*), and rabbit (*Oryctolagus cuniculus*) were obtained from the Laboratory of Animal Genetics, Breeding and Reproduction, Sichuan Agricultural University. For human subjects, we recruited healthy mothers and their infants at Sichuan Agricultural University who had not received antibiotics recently. The mothers’ ages at the time of milk sample collection were 25–32 years old. Fecal samples from their infants were collected. Fresh cow milk was used to feed newborn lions, while other animals in this experiment were fed maternal milk. We provided sterilized connectors and sterile bottles. For human participants, maternal milk was expressed into sterile bottles with a breast pump using sterilized connectors. Fresh feces of newborns needed to be quickly placed into a sterile collection tube, and then were stored in a −80 °C freezer until the nucleic acids were extracted.

### 2.2. DNA Extraction and 16S rRNA Gene Sequencing

Microbial DNA was extracted from the neonatal fecal samples using a QIAamp DNA Stool Mini Kit (Qiagen, Duesseldorf, Germany). Microbial DNA was extracted from the maternal milk using a DNeasy PowerFood Microbial Kit (Qiagen). Both procedures were carried out according to the manufacturer’s instructions, with minor modifications. The extracted DNA was quantified using a NanoDrop2000 spectrophotometer (Thermo Fisher Scientific, DE, USA), and the DNA integrity was determined by 1% agarose gel electrophoresis. Amplification of the V4 region of bacterial 16S r RNA genes was carried out as previously described [26]. In brief, barcoded universal primers 515F and 806R were designed for PCR amplification with initial denaturation at 95 °C for 5 min and 27 cycles of denaturation at 95 °C for 30 s, annealing at 55 °C for 30 s, and elongation at 72 °C for 45 s, followed by a final extension at 72 °C for 10 min. The PCR products were gel-purified, quantified via a NanoDrop™ 2000 spectrophotometer (Thermo Scientific), pooled at equal molar ratios, and sequenced on an Illumina HiSeq 2500 platform.

### 2.3. Sequence Analysis and Statistical Analysis

Raw sequence data from a total of 71 samples were processed using QIIME2. The DADA2 method was used for the sequence quality control. Barcode and primer sequences were removed from the 5’ ends and low-quality bases were truncated from 3’ ends according to the Q20 standard. The clean fasta sequences were overlapped, and then the feature table was constructed after de-redundancy. The final high-quality representative feature sequences were used for taxonomic annotation with the SILVA rRNA database (132_99 release) as a reference (https://www.arb-silva.de/ (accessed on 25 January 2021)). Shannon indexes and observed OTUs were computed to evaluate alpha diversity, and different animals were compared using the Kruskal–Wallis h-test. Weighted UniFrac and Bray–Curtis distances for each pair of samples were calculated to represent similarity relationships of the gut microbiota, and visualized using principal coordinates analysis (PCoA). PERMANOVA was used for group significance tests of beta-diversity. Significantly differential abundant feature detection of bacteria among groups were used in further linear discriminant analysis effect size (LEfSe) analysis (http://huttenhower.sph.harvard.edu/galaxy/ (accessed on 28 May 2021)). Phylogenetic Investigation of Communities by Reconstruction of Unobserved States (PICRUSt2) was used to analyze the functions of the maternal microbes. Pathways were predicted using the KEGG database. Differentially expressed pathways between carnivore and herbivore groups were analyzed with a Welch test using STAMP software (Version 2.1.3) (http://kiwi.cs.dal.ca/Software/STAMP, accessed on 22 November 2021). Differentially expressed pathways with a *p*-value < 0.01 are presented. To predict the source microbe of bacterial communities in neonatal feces, we used Source Tracker R package (Version 1.0) (https://github.com/danknights/sourcetracker, accessed on 22 November 2021) to compare sequences in breast milk as input samples (source samples) with the sequences in neonatal feces (sink sample). Statistical analysis was performed using SPSS 22.0 (SPSS, Chicago, IL, USA) and the R program.

## 3. Results

### 3.1. The Compositions of Microbial Communities in Maternal Milk and Neonatal Feces of Mammals

After quality filtering and assembly, 5,202,990 16S rRNA gene sequences were obtained from 36 maternal milk and 35 neonatal feces bacterial DNA samples from mammals during the lactation period (average of 70,703 and 75,933 sequences per maternal milk and fecal sample, respectively; Supplementary Materials: Table S1). Rarefaction curves representing Shannon diversity and observed OTUs calculated at the OTU level (Supplementary Materials: Figure S1A,B) reached a plateau, suggesting that the majority of microbial diversity had been sufficiently captured and the amount of sequencing data was somewhat reasonable.

**Figure 1:**
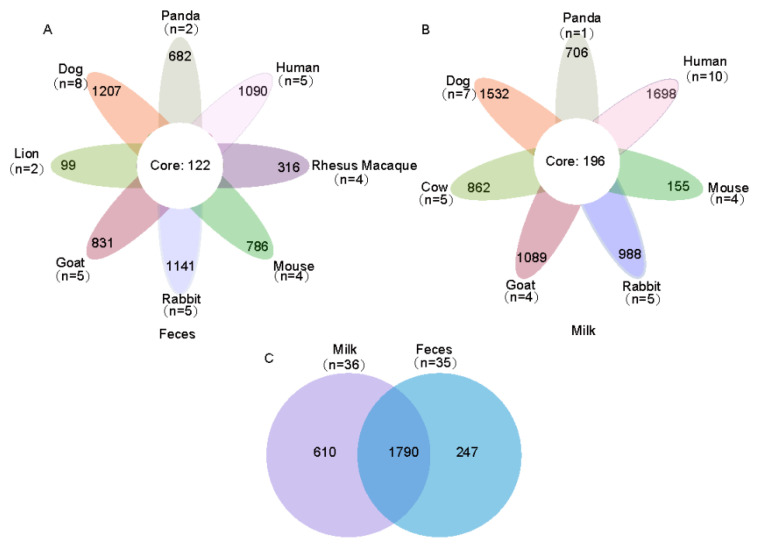
Flower plots showing the number of genera that were shared or not shared by: (**A**) neonatal feces; (**B**) the maternal milk of different species. (**C**) Core and unique bacterial populations of mammalian milk and neonatal feces at the genus level.

**Figure 2:**
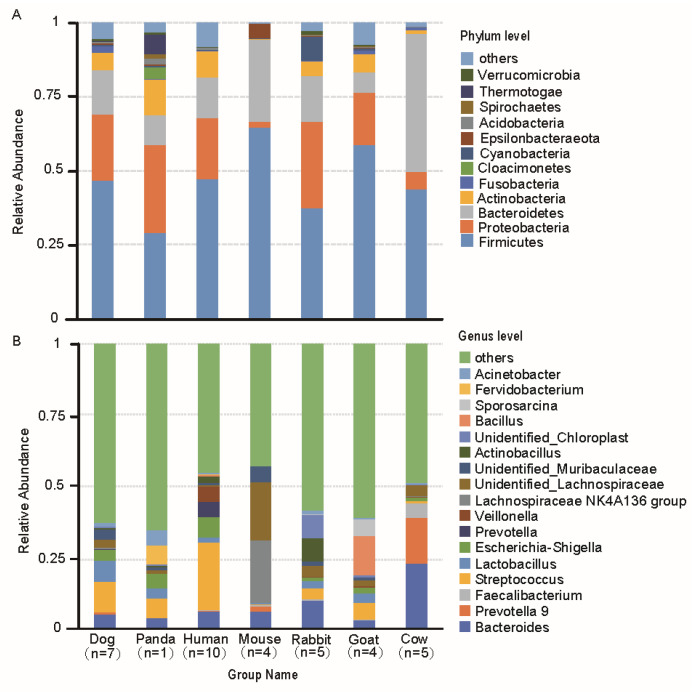
Maternal milk microbiome compositions in mammals. (**A**) The relative abundances of the top 12 (>1% of total sequences) phyla from maternal milk samples. (**B**) Bar graph of the top 17 (>0.05% of total sequences) genera from the samples. Each bar represents the average relative abundance of each taxon in the animals.

Sequences from maternal milk samples were classified into 55 phyla and 2640 genera. Neonatal fecal samples sequences were classified into 54 phyla and 2039 genera. In the maternal milk and neonatal feces microbes of mammals, the core microbiome examined had 196 and 122 shared genera, respectively. Moreover, maternal milk and neonatal feces shared 1790 genera microbiota (Figure 1A–C). The main microbiota compositions at the phylum (relative abundance > 1%) and genus (relative abundance > 0.05%) levels are shown in Figure 2A,B, respectively. At the phylum level, Firmicutes, Bacteroidetes, and Proteobacteria were the most dominant phyla in both the maternal milk and the neonatal feces of mammals (Figure 2A and Figure S2A). Other abundant phyla, including Actinobacteria and Fusobacteria, were found in the maternal milk of mammals (Figure 2A). At the genus level (with relative abundances > 0.05%), *Streptococcus* was the major genus in dog, panda, and human maternal milk samples (0.11%, 0.07%, and 0.24%, respectively), and *Bacteroides* was the major genus in maternal milk from goat and rabbit (0.23% and 0.10%, respectively). The mouse and goat maternal milk samples were largely dominated by the *Lachnospiraceae_NK4A136_group* (0.22%) and *Bacillus* (0.14%), respectively (Figure 2B). In addition, mammalian milk and neonatal feces shared eight domain genera, including *Streptococcus*, *Lactobacillus*, *Prevotella9*, *Bacteroides*, and *Faecalibacterium* (Figure 2B and Figure S2B).

### 3.2. The Diversity of Mammalian Maternal Milk and Neonatal Fecal Microbiota 

**Figure 3:**
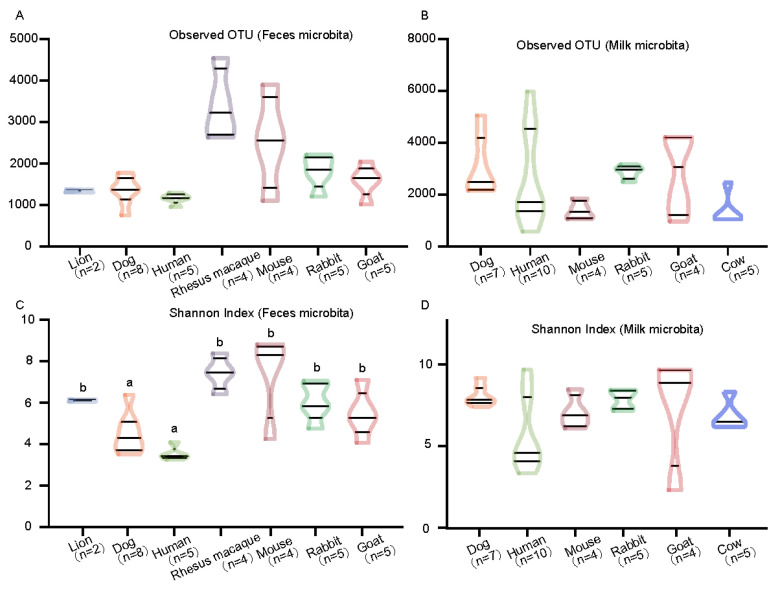
Comparisons of the diversity of the microbiota in mammals. The observed OTUs (**A**,**B**) and Shannon indexes (**C**,**D**) were compared using the Kruskal–Wallis test to determine significant differences.

The maternal milk and neonatal fecal microbiota of different species had different diversities in the observed OTUs and Shannon index (Figure 3). In the three type groups (herbivore, omnivore, and carnivore), the 16S rRNA data from feces samples indicated that the carnivores had a lower fecal microbial community diversity as measured by the observed OTUs and Shannon index (Figure S3A,B), while the omnivores had higher fecal microbial community diversity; however, alpha diversity was not significantly different between the carnivores, omnivores, and herbivores (Kruskal–Wallis, *FDR* > 0.01). The maternal milk microbial community diversity among animals showed the opposite result compared with neonatal feces (Kruskal–Wallis, *FDR* < 0.01).

**Figure 4:**
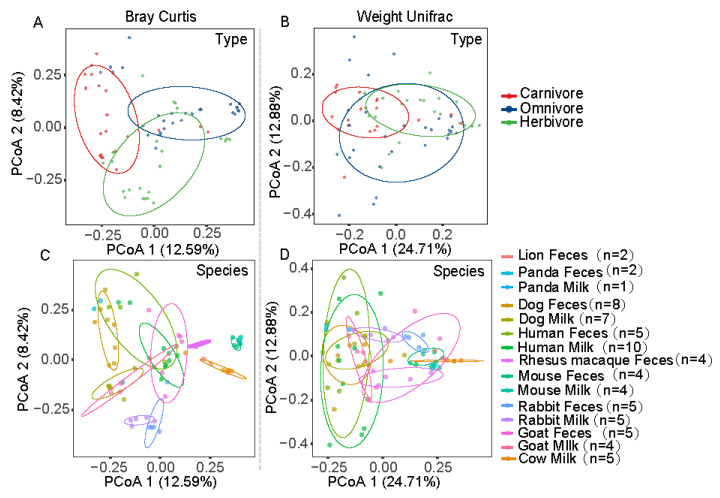
Principal coordinate analysis (PCoA) score plots based on the Bray–Curtis (**A**,**C**) and weighted UniFrac (**B**,**D**) distances, respectively. For the host taxonomic species and three type groups (herbivore, omnivore, and carnivore), the same data (samples) are shown in each panel. The percentage of the variation explained by the plotted principal coordinates is indicated on the axes.

Bray–Curtis-based principal coordinate analysis (PCoA) showed clustering by the three type groups (Figure 4A) and by taxonomic species (Figure 4C). The PCoA plot showed that the three type groups and a phylogeny shift in milk and fecal microbiota were evident in the carnivores, omnivores, and herbivores. However, the samples from lion feces did not cluster together with the samples from the other carnivores, even though lions are carnivores (Figure 4A). Lion fecal samples clustered more closely with cow milk samples than with other carnivore samples (Figure 4A). A similar result was obtained with weighted Unifrac analysis (based on the presence/absence of taxa) (Figure 4C,D).

### 3.3. Differences in Bacterial Communities and Functional Profiles of Maternal Microbiota

**Figure 5:**
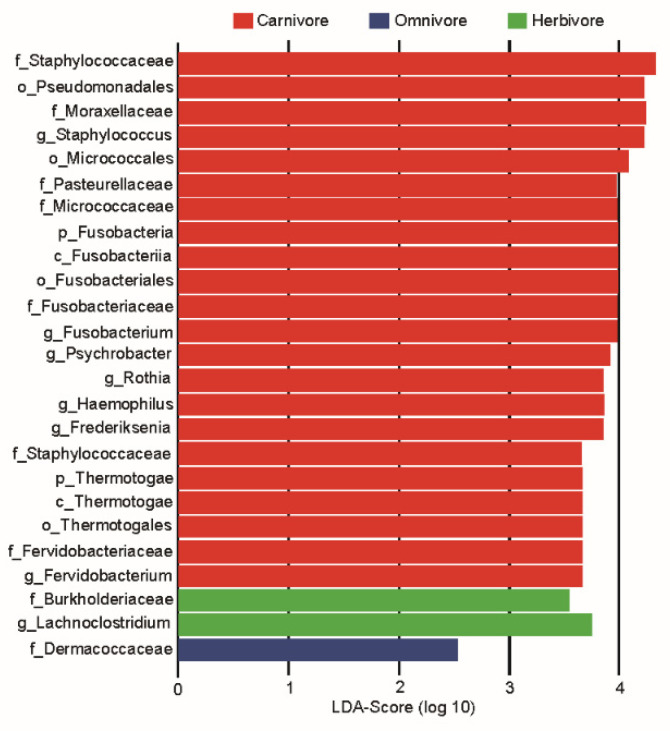
LEfSe of bacterial taxa showing significant differences among the three different type groups (herbivore, omnivore, and carnivore) (LDA score > 3.5).

**Figure 6:**
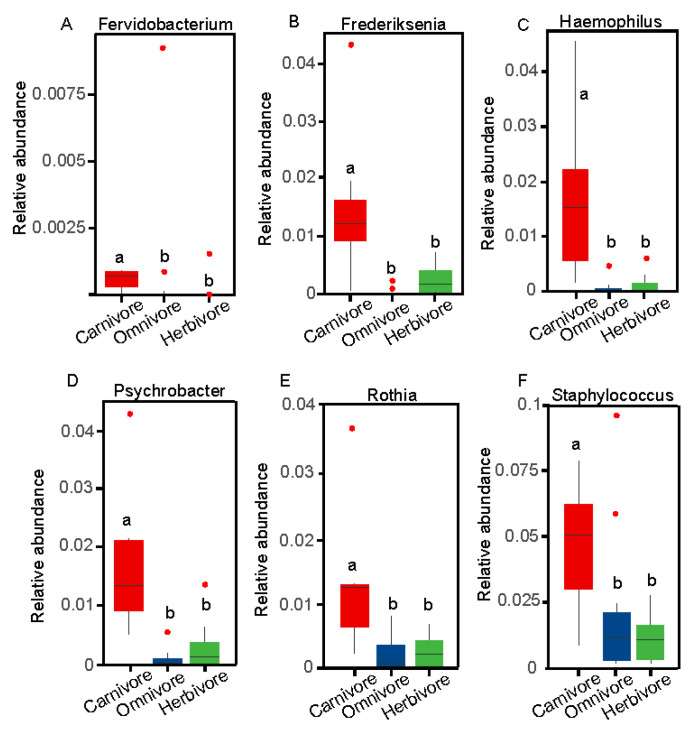
Boxplots showing specific differences in the relative abundances of discriminant genera(A-F) between the carnivore, herbivore, and omnivore maternal milk microbiomes (Wilcoxon rank-sum tests, FDR < 0.05).

To identify the significant abundant bacterial taxa in response to the three type groups (herbivore, omnivore, and carnivore), we used linear discriminant analysis effect size (LEfSe) to explore milk bacterial features (Figure 5). The omnivores’ maternal milk microbiota was enriched in *Dermacoccaceae*, while those of herbivores were enriched in *Burkholderiaceae* and *Lachnoclostridium*. At the family level in carnivores, *Staphylococcaceae*, *Moraxellaceae*, *Micrococcaceae*, *Fusobacteriaceae*, and *Pasteurellaceae* were dominant. At the genus level, the carnivore maternal milk samples were mainly characterized by higher abundance of *Staphylococcus*, *Fusobacterium*, *Psychrobacter*, *Rothia*, and *Haemophilus* (Figure 5). Additionally, we detected differences in the abundances of some microbes associated with different three type groups (Figure 6). For instance, *Fervidobacterium*, *Frederiksenia*, *Fusobacterium*, *Haemophilus*, *Psychrobacter*, *Rothia*, and *Staphylococcus* had the highest abundances in carnivores, but were significantly depleted in herbivores (Kruskal–Wallis test, *FDR* < 0.05).

**Figure 7:**
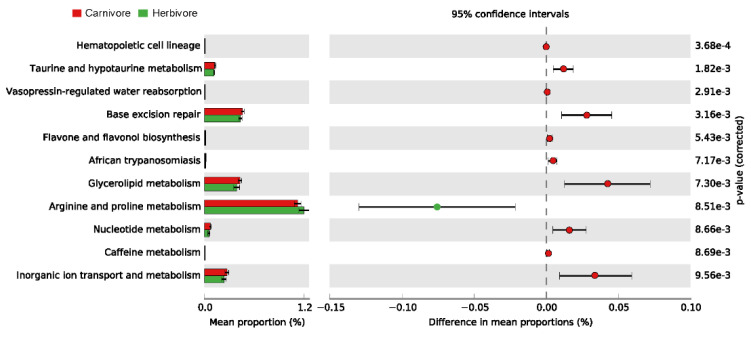
The bar plot shows the mean proportions of the differential KEGG pathways predicted using PICRUSt2. The difference in proportions between the groups is shown with 95% confidence intervals. Only *p*-values < 0.01 are shown.

Then, functional prediction by PICRUSt2 identified 11 different enrichment KEGG pathways between the carnivores and herbivores (Figure 7). The carnivore maternal milk had a significantly higher percentage of base excision repair, glycerolipid metabolism, taurine and hypotaurine metabolism, inorganic ion transport and metabolism, and nucleotide metabolism, while arginine and proline metabolism showed enrichment in the herbivore maternal milk. 

### 3.4. Neonatal Fecal Microbiota Composition: Association with Breastfeeding

**Figure 8:**
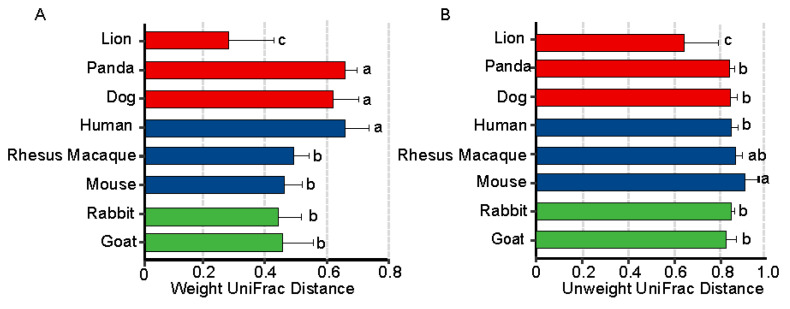

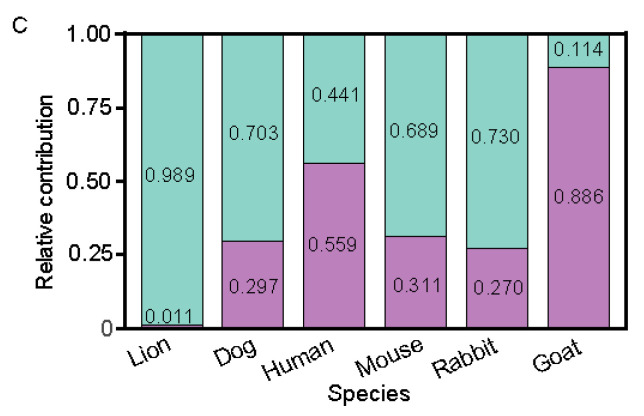
(**A**,**B**) Principal coordinate analyses based on weighted UniFrac and unweighted UniFrac distances. Bar plots labeled with different letters (a, b, and c) denote that the UniFrac distances between and within groups were significantly different (Kruskal–Wallis tests, *FDR* < 0.05). One bar represents the distance from the neonatal fecal sample of a species to the cow milk sample. (**C**) Relative contributions of maternal milk bacteria to neonatal fecal samples. The blue area represents the contribution of maternal milk bacteria to the neonatal fecal microbiota. The purple area represents unknown sources.

To analyze the influence of maternal milk on the assembly of the gut microbiota in neonates, we collected neonatal fecal and maternal milk samples. When we analyzed the microbial compositions of the maternal milk and neonatal fecal samples, we found that they shared many dominant bacterial genera (Figure 2B and Figure S2B). Moreover, PCoA based on Bray–Curtis distance showed that lion fecal samples more closely clustered with cow milk samples than with other carnivore samples (Figure 4A,C). We further compared the distances between neonatal fecal samples from all species and those from cow milk and maternal milk in this experiment; notably, the lion fecal samples clustered closest to the cow milk samples (Figure 8A,B and Figure S4A,B, *p* < 0.05), implying that maternal milk might serve as a microbial reservoir for vertical transmission.

To further determine the association between early gut microbial succession and the maternal milk microbiota, we collected neonatal fecal microbial samples from dog, lion, human, mouse, rabbit, and goat, as well as maternal milk samples for source-tracking analysis. The results showed that the contributions of maternal milk to the offspring of different animals differed (Figure 8C). Obviously, maternal milk from dog, lion, mouse, and rabbit were the primary contributors to the fecal microbiota, accounting for approximately 50% of the microbiota at day 3 after neonatal birth. However, the contributions of human and goat maternal milk to the fecal microbiota were less than 50% (human: 44.1%; goat: 11.4%). Interestingly, the contribution of maternal milk to the neonatal fecal microbiota also varied with the different three type groups. Overall, neonates received more bacteria from maternal milk (except for goat).

## 4. Discussion

It is well known that the gut microbiota is inextricably related to host health and physiological activities, and plays an important role in the process of maintaining host health. A large number of studies have aimed to explore the influence of host diet on the composition in and functional differences among the gut microbiota in mammals. Muegge et al. [4] analyzed fecal samples from dozens of mammals and found that both host diet and phylogenetic relationships influenced the diversity of the gut microbiotas. ln particular, the diversity of the gut microbiotas of hosts with different three type groups increased from carnivore to omnivore to herbivore. In our study, the carnivore group had a lower fecal microbial community diversity in neonatal fecal samples (Shannon index and observed OTUs), and the omnivore group had higher fecal microbial community diversity. However, the milk microbial community diversity in different groups showed the opposite result. This may be because the microbes in the maternal milk are not stable at the beginning of lactation [21]. In mammals, many factors influence the gut microbiome in early life, such as antibiotic treatment [27,28], breast milk [29,30], or environmental exposure [20] (including housing conditions [31], cohabitation with family members, [32,33], and geographical location [31]). Hongbin Liu et al. also found that the neonatal birth environment contributed 2–10% of mucosal microbiota in the large intestine within the first 2 weeks, and its contribution further diminished with age [34]. Therefore, breast milk and environmental exposure influence the neonatal gut at day 3 after neonatal birth. In addition, our sample size and the number of species may also have had a certain impact on the results.

The well-recognized functional data of the milk microbiota can be used to identify the quality of milk and the health status of mammary glands [35]. In this maternal milk microbial study, Firmicutes, Proteobacteria, and Bacteroidetes were the three most abundant phyla, similar to the results of several other studies that analyzed maternal milk samples from Western locations and China [36,37,38]. In our study, *Staphylococcaceae*, *Moraxellaceae*, *Micrococcaceae*, and *Fusobacteriaceae* had high LDA scores, indicating a high abundance in maternal milk, and should be used as an index for maternal milk. Pannaraj and colleagues [20] reported that *Moraxellaceae*, *Micrococcaceae*, and *Fusobacteriaceae* constituted the dominant families in human milk, and *Micrococcaceae* was the dominant lactic acid bacteria in goat milk. At the genus level, in our maternal milk samples, the dominant genera included *Streptococcus*, *Acinetobacter*, *Escherichia-Shigella*, *Bacteroides*, *Lactobacillus*, *Faecalibacterium*, and *Bacillus*. In addition to *Acinetobacter* and *Bacillus*, others bacteria were also very abundant in the neonatal gut samples. Similar to the results of the present study, *Streptococcus* and *Bacteroidetes* were the predominant genera in human milk and cow milk [39,40,41]. Others studies also reported *Staphylococcus*, *Pseudomonas* [18,42], *Acinetobacter* [43], *Leuconostoc* [44], and *Lactobacillus* as predominant genera. *Lactobacillus* and *Streptococcus* are the most common lactic acid bacteria genera in milk. *Bacillus* is widely used as probiotics [45]. As we know, probiotics provide a health benefit to the host [46]. *Bacteroides*, *Prevotella 9*, and *Faecalibacterium* are anaerobic taxa that are more typically associated with the gut microbiota. In our study, these three genera were the dominant microbes in the maternal milk and neonatal fecal samples, indirectly suggesting that microbes may be transmitted between mother and neonate. In addition, *Staphylococcus* is traditionally considered as a major mastitis-causing pathogen [47]. High bacterial abundance was not observed in any of the maternal milk samples, suggesting that the animal mammary glands may not have had signs of inflammation. However, it is important to note that *Staphylococcus* had a higher abundance in carnivores than in omnivores and herbivores. This suggests that carnivores are more likely to develop mastitis than omnivores and herbivores under certain conditions that cause imbalances in the species of bacteria that live in milk. Interestingly, we found that some of the bacteria, including *Fervidobacterium*, *Frederiksenia*, *Haemophilus*, *Psychrobacter*, *Rothia*, and *Staphylococcus*, differed significantly between the three different type groups. *Fervidobacterium* has the ability to utilize diverse complex carbohydrates [48]. Previously, *Rothia* was reported to be a harmful bacterium that causes neonatal bacteremia in compromised hosts [49]. Pathogenic *Haemophilus* is frequently transmitted from mother to neonate during breastfeeding [50]. *Psychrobacter* and *Moraxellae* are not considered to be important pathogens due to their short life span [51]. These bacteria are mostly found in high abundances in carnivores, suggesting that maternal milk microbes are influenced by the three type groups (herbivore, omnivore, and carnivore). Importantly, the majority of bacteria are pathogenic bacteria, so carnivore milk quality and glands may be affected if there are disruptions to these communities. 

In mammals, pig, cattle, and sheep produce proline from glutamine and glutamate in the small intestine [52]. In addition, proline plus hydroxyproline is most abundant in milk proteins. Proline plays important roles in protein synthesis and structure, metabolism (particularly the synthesis of arginine), and nutrition, as well as immune responses [53]. Arginine is extracted from the blood in amounts greatly exceeding the need for protein synthesis, and undergoes extensive catabolism in the gland, but about 20% of these amino acids is used for proline synthesis [54]. By predicting the function of maternal milk microbiota, the herbivore group had a significantly higher percentage of arginine and proline metabolism. It was indirectly indicated that maternal milk microbiota was involved in the metabolism of maternal milk. In tissues of developing animals and in milk, there are relatively high concentrations of taurine, which is the most abundant free amino acid in the body [55]. During fetal life or during suckling, specifically of the early milk, a deficiency in taurine manifests itself in fetal and postnatal growth deficits [56]. The glycerolipid metabolism pathway plays an important role in cellular metabolism and lactation maintenance, as glycerol can be converted into glucose by the liver and provide energy [57]. 

In this study, the newborns were fed maternal milk, except for the neonatal lion, which received cow milk. Interestingly, the PCoA based on Bray–Curtis distance showed that the lion feces samples more closely clustered with the cow milk samples than with the other carnivore group samples. The distances between the neonatal fecal samples from all species and cow milk were calculated in this experiment. A notable result was that the lion feces samples clustered closest to the cow milk samples, implying that the maternal milk and neonatal feces microbiomes might serve as microbial reservoirs for vertical transmission [58]. Finally, we characterized the bacterial communities in mother–infant pairs in six different mammalian species; the data suggested that bacteria from maternal milk were transferred to the neonatal feces.

The contributions of bacteria from maternal milk were highest at day 3 after neonatal birth in dog, lion, mouse, and rabbit (70.3%, 98.9%, 68.9%, and 73%, respectively). The contribution of bacteria from goat milk was low at day 3 after neonatal birth, but the contribution of bacteria from human milk accounted for 50% of the fecal bacteria in breastfed infants (goat: 11.4%; human: 49.1%). Obviously, maternal milk was the primary contributor to the microbiota in pig, accounting for approximately 90% of the microbiota during the first 35 days [34]. Another study [20] showed that primarily breastfed infants received more bacteria from human breast milk than those not primarily breastfed during the first 30 days of life (breast milk, 18.5% vs. 5.7%, *p* < 0.001, Wilcoxon rank sum). All these results suggest that maternal milk is the main source of microbes for neonates. However, the difference in the contribution of maternal milk microbes to the neonatal microbiome may be attributable to phylogenetic and physiological factors. For example, a simple stomach; degenerate cecum; and short, straight colon are typical in a carnivorous gastrointestinal tract [59]. Since the food (including milk) stays in the gut for only a short a time, more microbes from the milk are passed in the neonate gut. Most herbivores have evolved an elongated foregut or hindgut to prolong gut retention of indigestible food. The digesta is moved into the equivalent of the monogastric stomach after fermentation, and part of the microbiota is also digested; therefore, milk microbes are more likely to be excreted in carnivore feces [60]. Rabbit and goat are herbivores, and the contribution of bacteria from rabbit milk was higher than that from goat milk, which may be due to different physiological structures and body weights. Rabbits are smaller in size and have shorter guts than goats, and have only one simple stomach, while goats, as ruminants, have four stomachs. This could be why the microbes in rabbit gut were more similar to those in rabbit milk. There could be other reasons for this result, such as sample size or the number of species. Overall, maternal milk microbes were the major contributors to the gut microbiomes of neonates. The contribution of maternal milk microbes to the neonatal gut microbiota tended to be influenced by type (herbivore, omnivore, and carnivore). These results require additional studies with larger samples size for verification.

## 5. Conclusions

In summary, our study provided evidence of the effects of host diet and phylogeny on maternal milk microbiota. Maternal milk microbes were a major source of the neonatal fecal microbiota. The contributions of bacteria from maternal milk to the microbiota of neonates of different mammals was different at day 3 after neonatal birth. In short, our findings provided a theoretical basis for the maternal milk microbiota to affect neonatal fecal microbiota at day 3 after neonatal birth. 

## Data Availability

The sequencing data for this project were deposited in the National Genomics Data Center under accession number CRA004136.

## Supplementary Materials

The following are available online at https://www.mdpi.com/article/10.3390/ani11123349/s1, Figure S1: OTU-level alpha diversity of the maternal milk and neonatal fecal microbiota of mammals, Figure S2: Neonatal fecal microbiome compositions of the mammals, Figure S3: The observed OTU (A) and Shannon indexes (B) were compared using Kruskal–Wallis tests to determine significant differences, Figure S4: Principal coordinate analyses based on weighted UniFrac and unweighted UniFrac distances. Bar plots labeled with different letters (a, b, and c) denote that UniFrac distances between and within groups were significantly different (Kruskal–Wallis tests, FDR < 0.05). Table S1: rRNA Gene Sequences of Maternal Milk and Neonatal Feces of Mammals.
