# Supplementary figures and images for: The Maternal Milk Microbiome in Mammals of Different Types and Its Potential Role in the Neonatal Gut Microbiota Composition
Source: Animals (Basel). 2021 Nov 23;11(12):3349. doi: 10.3390/ani11123349 (PMC8698027; doi:10.3390/ani11123349)

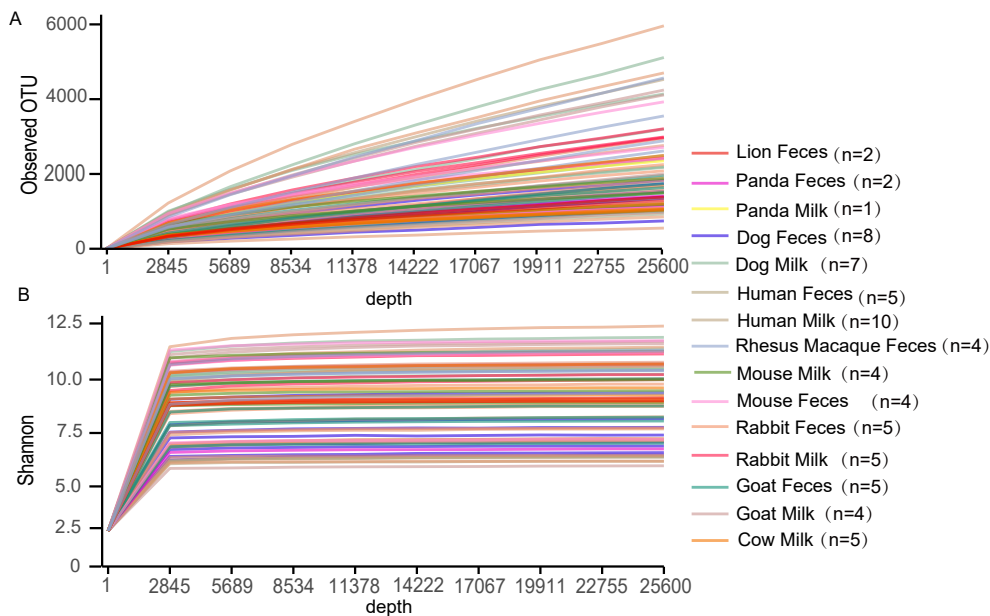

Supplement: Supplementary file 1 [file animals-11-03349-s001.zip › Supplementary/Fig S1.pdf]

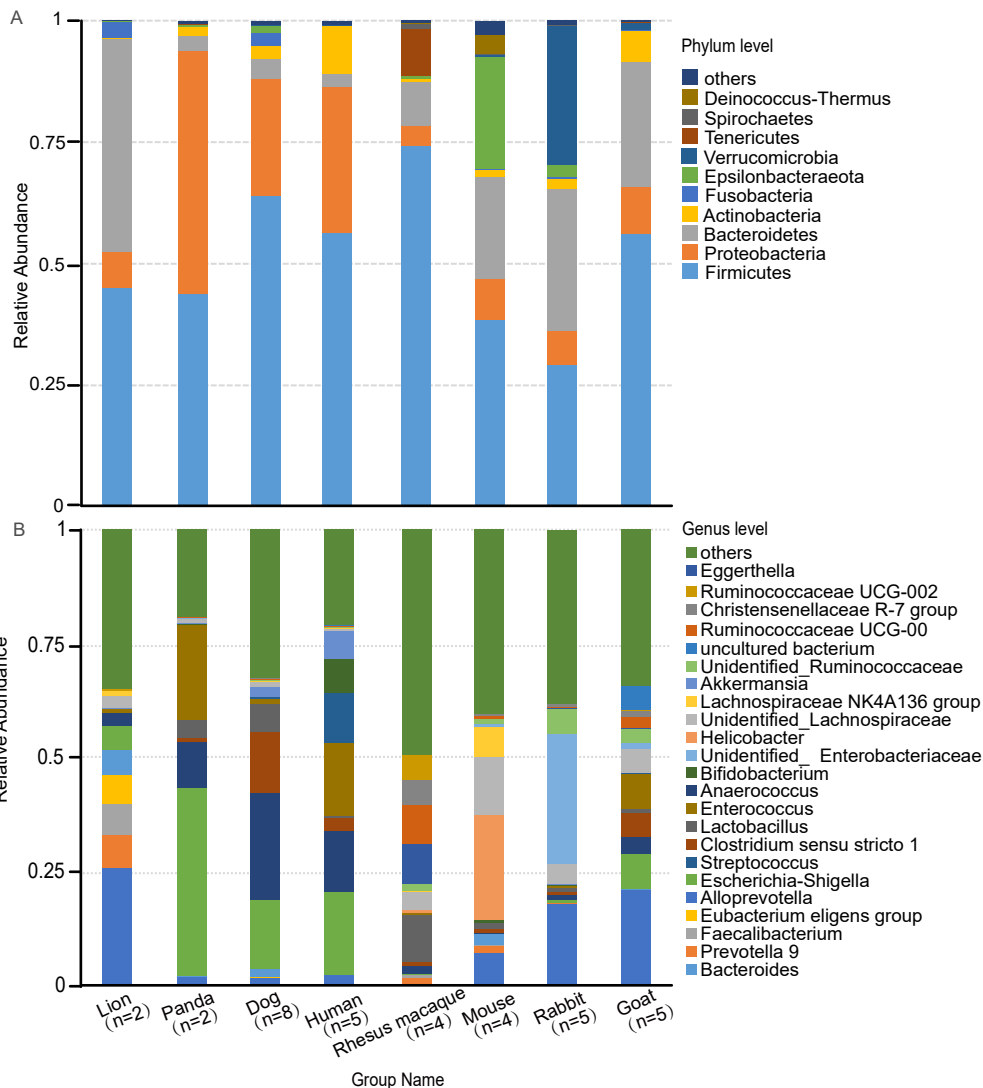

Supplement: Supplementary file 1 [file animals-11-03349-s001.zip › Supplementary/Fig S2.pdf]

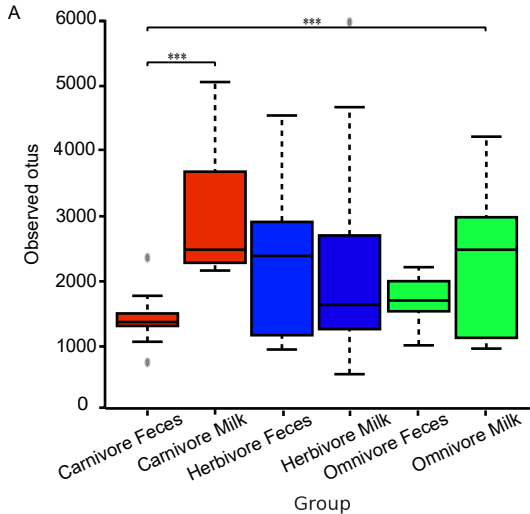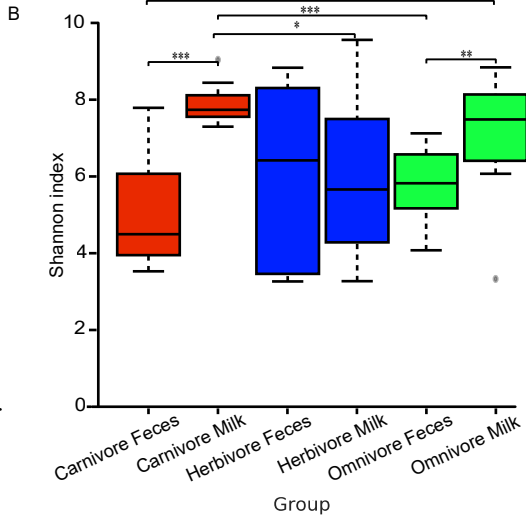

Supplement: Supplementary file 1 [file animals-11-03349-s001.zip › Supplementary/Fig S3.pdf]

A

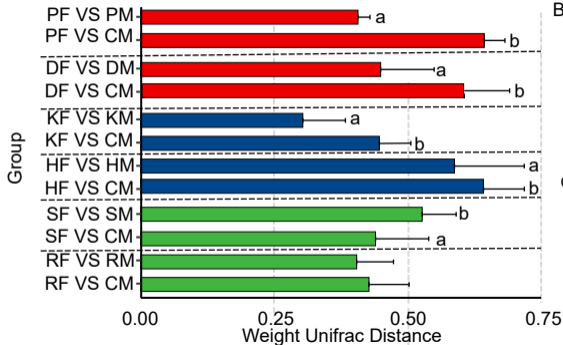

B

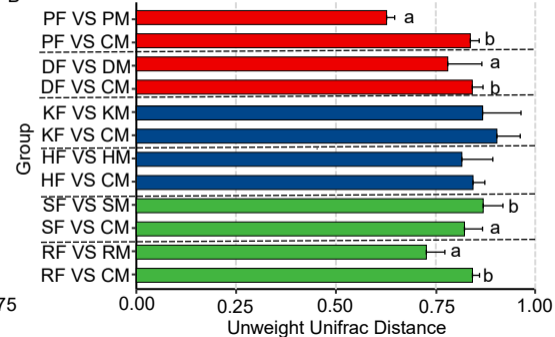

Supplement: Supplementary file 1 [file animals-11-03349-s001.zip › Supplementary/Fig S4 .pdf]
